# Little Things in Great Things

**Published:** 1898-05

**Authors:** 


ARTICLE IX.

### LITTLE THINGS IN GREAT THINGS.

Though we all look for great things, and for the most important features and most admirable qualities in great things, it is often the little things in great things and characters that are of the most importance, and the most important to know in judging of the whole value.

I was passing a mighty oak, when I spoke with much enthusiasm to a friend of its wonderful strength, its astonishing vitality, and its majestic proportions.

"Do you see yonder extremity of a twig curling its leaves?" said he. "Look carefully, and here and there you will see others. Observe also those little mites crawling in and out of the bark at places. These are little things compared with the general appearance of vigor; for the vast foliage and grand trunk speak of long life and still greater growth; but these little blemishes tell *me* as clearly as though I could look clear through it from root to top, that at heart it is rotten."

Only two years after this it fell with a great crash—not before a fierce wind, but in a storm of moderate force. It was rotten from center to sap wood.

The inspector of a great bridge, newly made, reported every thing "admirable." Its mighty beams, its strong trusses, its wonderful spans, and its massive cables were the pride of all observers.

A young man fishing from one of the huge chains, underneath the road bed, noticed a flaw in one of its links. It was only a slight opening, and yet it might become worse. Thought he, "A chain as a character, is only as strong as its weakest part, and though this is a little weakness among the great chains and cables and timbers of this bridge, it should be known." He therefore reported it. He was laughed at for his pains. But, by and by, the rattle and strain of an extra load snapped it, and away went the whole structure.

In our work as dentists, the greatest essentials are the little details, both because they are more liable to be overlooked and because all our most important operations are made up of these little details, and must be judged for better or for worse, by their character. If these are all well wrought the whole is perfect, but no little thing may be overlooked with impunity. The great Atkinson was once filling a frail central incisor when I saw a slight check in the enamel from the pressure of the gold. It required a magnifying glass to discover it with surety. "O," said he, "that is nothing," and he finished the beautiful filling that had cost him two hours' labor, and the patient much fatigue and twenty-five dollars. I saw it six months afterward, and sure enough it was but very slightly worse. But in a year the tooth was ruined by it.

And so in all the affairs of life. If we are careful of the little things of our appearance, and manners, and conduct, the great things will take care of themselves. A small blemish in the countenance mars the whole beauty. A little vulgarity, even from one looking clean and wholesome, is a stench in our nostrils. Dirty nails, unkempt hair or slovenly dress in a dentist, otherwise esthetic, is intolerable. "I went to young Brown the other morning to have my teeth filled," said a lady, "because I had learned to like him for his neatness, politeness and skill. But I had hardly taken the chair before the stench of stale tobacco smoke so offended me that I made an excuse for a postponement, and cannot make up my mind to return." And yet that young man had cleaned his mouth and attempted to disguise his breath with spices. "It is the little foxes that spoil the vine."

We are quite apt to reply when criticised :--

"Well, now, that is a very little thing. It is hardly worth mentioning."

Yes, it may be a little fault; and yet it is from these little faults that great faults begin.

When young, I was passing a high dam, and saw a little trinkle of water oozing out of the foundation. I began to play with it, and to direct its course. A gentleman with me in surprise said :—

"Quick! We must report this to the miller, or the whole dam will be ruined."

"O shaw," I replied, "I have seen it coming through here before to-day. I can stop it with a handful of clay."

But I had hardly taken my hand off before it burst through the clay, and brought with it little pebbles, and dirt from the dam. Even plugging it did no good; nor a great crib of cement. It only increased after such temporizing. The great pond itself had to be emptied, and the huge wall torn out where the leak was, before permanent repairs could be made.

Thus, when we discover even little faults in our character —or have them pointed out to us by others, friend or foe—we cannot repair them too soon, or too thoroughly, or at too great an expense. We must overcome them at all hazard, or they will overcome us.

"He is a fine fellow—but." But what? Whatever it is why should your friend cling to it?

How often I have heard patients say: "He is a good dentist—but."

How foolish to hold onto anything that is a *but* in our character or practice!

Said a fine young lady to me recently :—

"George is a smart young man—*but*." Why should that young man have any habit that should be in the way of his entire acceptability to the most esthetic young lady?

And what is our *but?* O for some friend or foe to show it to us; and then for courage to overcome it! We are surely growing better or worse; and as surely as none of us grow strong and skilful, and of sterling worth suddenly, so surely may we degenerate so slowly and by such "little things" that we are blinded and bound and destroyed by oue degeneracy. —*Dental Brief*.

